# How to Look Well

**Published:** 1891-02

**Authors:** R. A. Chudleigh


### HOW TO LOOK WELL.

Let it be understood, once and for all, that perfect health is the best and only true cosmetic. The proper amount of food to meet the requirements of the system should always be taken ; there is no beauty or attraction in that state of wan ghastliness which comes from insufficient nutrition. The use of the pores is to keep the system pure and healthy; they should be aided rather than retarded in their work. But what chance do they have, for instance, to keep the surface of the face at its best, when their delicate mouths are buried under a glutinous coat of oil, glycerine, or other tenacious base, upon which a mass of "powder" has been deposited ? There is no need for an answer. Perspiration is the most natural and effective method of freeing the pores of the hardened secretions which so often form "blackheads" and in other ways mar the complexion. Artificial perspiration, in cases where its necessity is apparent, may best be secured by the Turkish bath, if that is available. If a substitute must be had, a formula which recently appeared, gives good promise. To begin with, the face should be washed with water as warm as can be comfortably borne, and given gentle friction with a rubber glove or a good firm bath towel, but only while the skin is wet. While rubbing, keep a pot of pure olive oil handy, and smear a little on the face during the operation. Dry the skin carefully, but add neither powder nor lotion, then get into a hot bath, and with a flesh-brush and a little soap make the whole body in a warm heat and add hot water to keep up the full heat during the process. Dry quickly, wrap in a woollen night-gown, and at once get into bed, and cover with several blankets. Drink also about half-a-pint, or even more, of quite warm lemonade, made from freshly cut lemons. This will create a nice, gentle perspiration, and after a few nights this will increase, and the pores will free themselves in a natural manner. In the morning, before washing, apply a little lemon juice where the pores are affected, and rub gently with a towel. Once the pores are free it is easy to keep them so.

Black specks on the nose and chin are almost certainly comedones, for which "a certain cure" will be sought in vain. Not only, as a matter of fact, have the remedies always failed, but there also seems to be a good reason why they should fail, viz., that the comedones are not so much a disorder as a natural character of those portions of the skin of the face on which the hairs are almost or quite suppressed. We are all of us potentially hair-clad animals—either as hair or as down our shaggy coat is discernible over nearly our whole surface, and the trend of the hair follows an invariable and very definite plan. But as in many other animals, so in ourselves, there seems to be a tendency to a suppression of the hair on the nose, and in a less degree on sundry other regions about the mouth and face. But, although the hairs be suppressed, yet the follicles, or sockets, in which the hair should grow continue to exist. Moreover, the sebaceous glands which secrete the natural hair-oil continue to be developed in the follicles. Now, if these glands be obstructed or irritated they secrete a more solid and more tenacious lubricant than is natural, and as there is no hair to push it out the follicle speedily becomes choked ; the orifice becomes dark with dust, and the result is a comedo. By simple pressure the comedo can be made to evacuate its lardy contents in the form of a small pale worm with a dark head. Hence comedones are often called flesh-worms, which they are not, though the microscope shows that, like most other unoccupied corners, these disused hair-follicles are tenanted by some perfectly harmless parasites about 130th of an inch long, looking not unlike a minute chrysalis. Though not wholly removable, these follicles can be rendered inconspicuous by keeping them cleaned out. This may be effected to some extent by pressing out their contents ; but as this leaves an orifice which catches dirt and is coarse in appearance, it is preferable to soften the skin with emollient applications, to cleanse it with a steam bath or warm fomentations, and finally to stimulate it with gentle friction not carried to the extent of causing a raw or excoriated sensation. When the skin has regained somewhat of its normal appearance, it is better to discontinue all external applications and rely on periodic massage, that is, pressing and rubbing with the fingers twice or thrice a day, using warm water and a superfatted soap. —R. A. Chudleigh, *in Farm and Home.*

